# The Association Between Suicidal Behavior, Attentional Control, and Frontal Asymmetry

**DOI:** 10.3389/fpsyt.2018.00079

**Published:** 2018-03-14

**Authors:** Catherine Thompson, Elsie Li Chen Ong

**Affiliations:** ^1^School of Health Sciences, University of Salford, Salford, United Kingdom; ^2^Division of Information and Technology Studies, Faculty of Education, The University of Hong Kong, Pokfulam, Hong Kong

**Keywords:** suicide, attentional control, inhibition, frontal asymmetry, emotional Stroop, capability model

## Abstract

It can be difficult to identify those at risk of suicide because suicidal thoughts are often internalized and not shared with others. Yet to prevent suicide attempts it is crucial to identify suicidal thoughts and actions at an early stage. Past studies have suggested that deficits in attentional control are associated with suicide, with the argument that individuals are unable to inhibit negative thoughts and direct resources away from negative information. The current study aimed to investigate the association of suicidal behavior with neurological and behavioral markers, measuring attentional bias and inhibition in two Stroop tasks. Fifty-four participants responded to the color of color words in a standard Stroop task and the color of positive, negative, and neutral words in an emotional Stroop task. Electroencephalographic (EEG) activity was recorded from frontal areas during each task and at resting. Participants were separated into a *low-risk* and *high-risk* group according to their self-reported suicidal behavior. Participants in the high-risk group showed slower response times in the color Stroop and reduced accuracy to incongruent trials, but faster response times in the emotional Stroop task. Response times to the word “suicide” were significantly slower for the high-risk group. This indicates an attentional bias toward specific negative stimuli and difficulties inhibiting information for those with high levels of suicidal behavior. In the emotional Stroop task the high-risk group showed reduced activity in leftward frontal areas, suggesting limitations in the ability to regulate emotional processing via the left frontal regions. The findings support the argument that deficits in attentional control are related to suicidal behavior. The research also suggests that under certain conditions frontal asymmetry may be associated with suicidal behavior.

## Introduction

Suicidal behavior refers to a wide range of suicide-related cognitions, emotions, and behaviors (1). It is a term that has been used to categorize behavior associated with ideas, intentions, motivations, plans, and attempts for suicide. Prediction and prevention of suicide is challenging because it is a personal and sensitive topic (2). Those who experience suicidal behavior may avoid discussing this with others and sharing their thoughts can often trigger feelings of stigmatization. This can also lead to difficulties in identifying those who are vulnerable to suicide because assessments are largely based on clinical interviews and self-report measures (3). This means clinicians have to rely on individuals self-disclosing information regarding their current suicidal thoughts and plans, and any history of past suicide attempts. Such disclosure may be unreliable if a person is unwilling to report their intentions (4) and individuals may deliberately deny or conceal their suicide tendency to avoid intervention or hospitalization (2, 5). This highlights the importance of developing alternative measures for identifying individuals with a suicide risk. One potential option would be to use measures of cognitive and neurological processing (5).

Deficits in cognitive processing and neurological activity have been found in suicidal individuals and these are specifically related to “executive function” (6–9). Executive function [also termed cognitive control, e.g., Ref. (10)] constitutes many components that allow an individual to plan and execute goal-directed behavior including the ability to regulate emotions, exert inhibitory control, shift focus between multiple tasks, and flexibly modify behavior according to a situation (11–14). Deficits therefore relate to impairment of a broad range of cognitive functions such as memory, attention, and decision-making.

Miyake et al. (15) identify three key components of executive function: shifting, updating, and inhibition. Updating is the ability to maintain relevant information within working memory and to update this information in accordance with changes in task demands. Shifting refers to directing attentional resources away from task-irrelevant information and toward task-relevant information. Inhibition is the ability to override an automatic but irrelevant response. Attentional control (the ability to flexibly shift attentional resources in dynamic situations, maintain focus on relevant information, and inhibit irrelevant information) is implicated in each of these components. Deficits in attentional control are argued to be related to affective disorders as individuals have difficulties shifting resources away from negative thoughts and re-directing attention toward more positive information (16). The importance of executive control in the development and maintenance of affective disorders is outlined in an integrative cognitive-biological model of depression that proposes two key components (17, 18). Initially, low-level bottom–up processing of negative information results in attentional biases. Second, deficits in top–down control processes mean that an individual is unable to direct attention away from negative information.

Executive dysfunction is argued to have a direct impact on emotional regulation as it prevents individuals engaging in effective mood-regulation strategies and instead a person may utilize maladaptive strategies that serve to sustain negative biases. For instance, Joormann and Tanovic (10) make the argument that individuals with major depressive disorder may have difficulties changing the contents of working memory and moving the focus away from negative thoughts (updating). Deficits in shifting have also been associated with increased rumination in depressed individuals due to an inability to shift the focus of attention away from negative thoughts [e.g., Ref. (19)]. This increased focus on negative information serves to enhance and maintain negative mood states (20).

While the theoretical explanations for the links between executive function and affective disorders focus on depression, deficits in executive control have also been linked to the reduced ability to deal with emotional disturbances that are commonly found in suicidal patients (21, 22). For example, difficulties with response inhibition can make one more likely to act impulsively, while impairments in interference control can prevent the inhibition of irrelevant and intrusive thoughts, such as those relating to self-harm (13, 23). Richard-Devantoy and Courtet (24) proposed that suicidal individuals with impaired executive function are at a greater risk of attempting suicide due to their diminished ability to engage in protective cognitive strategies. This is because they are less able to accurately assess the consequences of their behavior, and less capable of inhibiting maladaptive emotional and behavioral responses. They found that individuals who had attempted suicide showed deficits in decision-making, problem solving, autobiographical long-term memory, and working memory. Loyo et al. (25) measured the links between executive functioning and suicidal behavior, taking measures of attentional control, abstract reasoning ability, and decision-making in 25 suicide attempters with depressive symptoms, 25 non-suicide attempters with depressive symptoms, and 24 non-depressed participants. Consistent with Richard-Devantoy and Courtet (24), they found that compared with the non-suicide attempters and non-depressed participants, suicide attempters showed greater deficits in a range of tasks including the Wisconsin Card Sorting Task and the Iowa Gambling Task. These findings suggest a relationship between executive dysfunction and suicidal behavior. Interestingly, a study by Keilp et al. (16) that compared executive function in depressed suicide attempters, depressed non-attempters, and healthy controls found that while the patient groups showed poor performance across a number of measures, those with a past suicide attempt showed specific deficits in tests of attentional control and working memory.

Further supporting evidence for the link between suicide and executive control comes from a study by Richard-Devantoy et al. (26) using a color-word interference test similar to the Stroop task. Participants were 17 healthy controls and 38 depressed individuals with no suicide attempts or ideation (thoughts of suicide), 16 depressed individuals with suicide ideation, 14 depressed low-lethality suicide attempters, and 17 depressed high-lethality suicide attempters. The task involved color naming, word reading, inhibition, and inhibition/switching trials and compared with healthy controls and those with suicide ideation, high-lethality suicide attempters took longer to respond to inhibition trials. Richard-Devantoy et al. (26) argued that the results have important implications for suicidal behavior because deficits in executive control may undermine the ability to deal with real-life emotional distractions. While individuals with adequate inhibition may exert control over inappropriate behaviors (such as self-harm) and are better able to resist suicidal urges, those with impaired inhibition may be less able to exercise control over these impulses, and may have difficulty resisting the urge to act on suicidal thoughts. Such deficits may therefore predict whether an individual will engage in suicidal behavior. The authors do however state that executive control is impacted by age and their findings are limited due to the fact that they used a group of older adults. Consequently it would be beneficial to assess executive dysfunction in a younger population.

Executive function is primarily controlled by the prefrontal cortex (PFC), which interacts closely with other brain regions such as the anterior cingulate and the amygdala (13, 27). It has been proposed that the frontal regions of the brain, predominantly the dorsal lateral and ventral lateral PFC (dlPFC, vlPFC), are responsible for guiding attention, maintaining information within the mind, shifting cognitive resources between different sources of information, and inhibiting the processing of task-irrelevant information (8, 9, 28–30). Compton et al. (28) measured PFC activity using functional Magnetic Resonance Imaging (fMRI) in a color Stroop and an emotional Stroop task. In a color Stroop task (31), participants are asked to name the color of words that possess a congruent (e.g., the word red printed in red) or incongruent semantic meaning (e.g., the word red printed in green). In an emotional Stroop task (32), participants are asked to name the color of emotional and neutral words. Both tasks require the inhibition of an automated, irrelevant response (reading the word), and the allocation of resources to process a relevant response (the name of the color); therefore, they allow for the measurement of attentional control. In general, studies show that responses are slower to incongruent trials compared with congruent trials in a standard color Stroop, revealing difficulties inhibiting the automatic processing of irrelevant information [known as the Stroop interference effect (33)]. Responses are also slower to emotional words compared with neutral words in an emotional Stroop task [also known as the emotional Stroop effect (34–36)]. This is particularly the case in patient groups when the emotional words are related to affective disorders [e.g., Ref. (37)]. Compton et al. (28) found that activity in the dlPFC increased for trials in which the word was incongruent to the color and for trials in which negative words were presented. It is argued that increased activity reflects greater investment of resources in order to inhibit automatic responses (regardless of whether these are emotionally significant); therefore, difficulties recruiting the dlPFC would lead to impaired inhibition.

Pan et al. (38) measured response inhibition using the Go/No-go task in adolescents with various degrees of suicidal thoughts and actions. The sample included 15 depressed adolescents with a history of suicide attempts, 15 depressed adolescents with no history of suicidal behavior, and 14 healthy controls. The Go/No-go task requires participants to press a button in response to a target stimulus (Go), but to inhibit the button press and do nothing in response to a non-target stimulus (No-go). In the healthy controls, fMRI recordings showed increased activation of the prefrontal, anterior cingulate, and parietal cortical regions. The anterior cingulate in particular is considered crucial for inhibitory control (8) and while the depressed individuals with no past history of suicidal behavior did not differ from the controls with regard to activity in this area, the depressed adolescents with a history of suicide attempts showed significantly reduced activity. This indicates impairments in inhibitory control for suicidal individuals and also shows the relationship between cognitive processing and cortical activity. The findings were consistent with those of Compton et al. (28) regarding the association between frontal activity and attentional control. They also suggest that this association may constitute a neurobiological basis for predisposition to suicidal behavior. It is proposed that executive control may moderate frontal cortical activity and neurological measures may therefore be used to predict suicidal behavior of individuals beyond the currently used self-report measures.

The majority of past research exploring the neurological basis of affective disorders has focused on clinical depression and there are comparatively fewer studies that measure executive function and neurological activity in suicidal populations. The initial argument that limited executive function and patterns of PFC activity may be related to affective disorders came from observations of patients who had experienced a stroke and were suffering from clinical depression [(39); see Ref. (40) for a review]. It was evident that following damage to the left prefrontal regions some patients became increasingly depressed, while damage to the right frontal regions resulted in increasing levels of manic symptoms. Schaffer et al. (41) explored this dissociation by measuring cortical activity in patients who were suffering from depression to varying extents. The aim was to identify any “asymmetry” of activity to support the claim that different patterns of frontal activation may be related to the severity of the disorder. Electroencephalogram (EEG) electrodes were placed on the frontal and parietal regions of the brain and similar to the clinical report of Gainotti (39), patients indicating more severe symptoms of depression showed greater activity in the right compared with the left. Importantly, this pattern was only found in the frontal regions, not the parietal regions.

These findings led to a rapid expansion of research surrounding lateralized frontal activation and EEG has been a common tool used to measure the correlates of relative hemispheric dominance (42, 43). It has been posited that the left hemisphere (LH) is dominant for processing positive emotions, whereas the right hemisphere (RH) is dominant for processing negative emotions. This means that if individuals have greater electrocortical activity in the right frontal region, they will have a disposition toward focusing on negative emotions and information. Supporting evidence for this came from Davidson and Fox (44) who were among the first to use asymmetric frontal cortical activity to make inferences about frontal asymmetry and emotions. They suggested that patterns of lateralized brain activity can be identified as early as infancy and to test this hypothesis they recruited 10-month old infants to view videotapes consisting of happy or sad facial expressions. Activity in frontal and parietal regions was recorded and it was found that increased activation in the left (relative to the right) corresponded to viewing happy faces while increased activation in the right (relative to the left) corresponded to viewing sad faces. The findings were also consistent with those of Schaffer et al. (41) as the differential pattern of activity was only found in the frontal regions, not the parietal regions.

Based on this research, Davidson et al. (45) and Davidson et al. (46) developed the *dispositional model*. The model holds a valence hypothesis that positive affect is associated with leftward frontal cortical activity, and negative affect is associated with rightward frontal cortical activity [e.g., Ref. (42)]. Since the introduction of this model research has been conducted to show the relationship between asymmetric frontal activation and depression (42, 43, 47–50). Overall the findings show that patients with a history of depression or with recurrent depression have relatively lower left frontal cortical activity (51), also known as left frontal hypoactivation [for reviews, see Ref. (52–54)]. This is in contrast to healthy controls that show the opposite pattern with greater leftward frontal cortical activity (50, 55). The level of reduced leftward activity also correlates with the level of symptoms reported, suggesting that this may provide a potential marker for assessing severity of disorders in patients (56).

The dispositional model makes the assumption that positive emotion is always associated with leftward frontal activation and negative emotion is always associated with rightward frontal activation. This has been challenged by Coan et al. (57) who proposed the *capability model*. This model supports the claim that individual differences in frontal asymmetry exist but argues that the differences will vary according to different situational contexts (40, 58). Therefore, while the dispositional model posits that rightward frontal activity will correspond to more negative emotional responses in all situations (e.g., in events that trigger joy, fear, or sadness), Coan and Allen (58) propose that differences in frontal asymmetry correspond to the different emotional demands of a situation. The capability model therefore suggests that asymmetrical differences are best thought of as interactions between individual differences and situational demands.

Despite the differences in these two models, neurological findings provide evidence that frontal asymmetry may serve as an indirect neurological indicator for predicting depression, or even suicide risk. For instance, using event-related fMRI, Jollant et al. (59) compared the neural activity of previously depressed men with past suicide attempts, previously depressed men with no suicide attempts, and healthy male controls. Across the three groups, only those with a history of suicide attempts showed frontal asymmetrical differences in response to emotional faces (angry, happy, and neutral). Specifically, they showed increased neural activation in the right lateral orbitofrontal cortex in response to angry faces relative to neutral faces. Jollant et al. argue that increased sensitivity to another person’s disapproval (e.g., in the form of an angry facial expression) and a higher propensity to process and act on negative emotions may exacerbate suicidal behavior in suicidal individuals. This links to the proposal that increased processing of negative information (as demonstrated by increased activation) may serve to maintain negative attentional biases in individuals suffering from affective disorders (10, 17, 18).

Grimshaw and Carmel (60) provided an explanation for the inhibitory difficulties in depressed individuals arguing that they are unable to utilize the parts of the brain (i.e., the left PFC) responsible for inhibition, particularly the inhibition of negative information. Studies have supported this by showing that failure to recruit the left dlPFC when presented with irrelevant negative information is associated with depression (61, 62) and trait negative affect (63). Given the relationship between frontal asymmetry and inhibition, Grimshaw and Carmel (60) have proposed the *asymmetric inhibition model*. It is predicted that each frontal region specializes in the inhibition of different types of emotions, with the left dlPFC responsible for inhibiting negative stimuli, and the right dlPFC responsible for inhibiting positive stimuli. Therefore, frontal asymmetric activation reflects the ability to inhibit different types of emotional stimuli.

The current study aims to further investigate the relationship between frontal asymmetry, executive function (specifically attentional control), and suicidal behavior. While the majority of the previous research focuses on clinical samples there is an argument that early identification of those at risk of suicidal behavior is essential (64, 65). On the basis of this, the present work explores the links between suicidal behavior, attentional control, and asymmetry using a non-clinical population reporting relatively mild symptoms. Frontal asymmetry was recorded from individuals reporting high and low levels of suicidal behavior at resting state (both eyes closed and eyes opened) and during a color Stroop task and an emotional Stroop task. The dispositional model (45, 46) asserts that individuals who report higher levels of suicidal thoughts and behaviors will exhibit rightward frontal activity compared with those with low suicide risk regardless of the situation. However, the capability model (57) argues that the effect of suicidal behavior on asymmetric frontal brain activation will be more pronounced during emotionally demanding situations. By comparing frontal asymmetry at resting state and in emotional and non-emotional tasks it will be possible to test the predictions of these two models. Using the Stroop task also allows differences in attentional control to be compared according to levels of suicidal behavior. It is proposed that individuals reporting higher levels of suicidal behavior (high-risk) will show more difficulties in attentional control and will therefore be at a greater risk of suicide (and more likely to make a future suicide attempt) because they are less able to inhibit negative thoughts and direct attention toward task-relevant information. In contrast, those who experience low levels of suicidal behavior will have effective attentional control and will therefore be less likely to focus on irrelevant negative thoughts and actions. On the basis of this it was predicted that individuals with a high-risk would show a bigger Stroop interference effect in the color Stroop task compared with those in the low-risk group. For the emotional Stroop task, it was predicted that all participants would show the expected emotional Stroop effect, but that the high-risk group would show increased difficulty inhibiting negative words. According to the models of frontal asymmetry it was hypothesized that those who report high levels of suicidal behavior would also show relatively higher rightward frontal activation during the color Stroop task. Additionally, in the emotional Stroop task, leftward frontal activation would correspond to inhibition of negative stimuli whereas rightward frontal activation would correspond to inhibition of positive stimuli.

## Materials and Methods

### Design

The study used a mixed measures design to investigate the effects of suicidal behavior in a Stroop task and an emotional Stroop task. Suicidal behavior was a between-participants variable with two conditions, high-risk and low-risk. In the color Stroop task a 2 (suicidal behavior) × 2 (congruency) design was used. Congruency referred to whether each color word was the same (congruent) or different (incongruent) to the color of ink in which the word was presented and this was a within-participants variable. In the emotional Stroop task a 2 (suicidal behavior) × 3 (emotion) design was used. Emotion was the valence of the words presented with positive, negative, and neutral words. This was a within-participants variable. The dependent variables were accuracy (total number of correct responses), and response times (milliseconds) to respond to the color of each word. A self-reported measure of depression was also recorded for each participant.

Frontal asymmetry (uV^2^) was recorded during resting state and during the color Stroop and emotional Stroop tasks. In the resting state and color Stroop task asymmetry was compared between the high- and low-risk groups. In the emotional Stroop task asymmetry was compared between these two groups and across the three conditions of emotion.

### Participants

Fifty-four undergraduate students (32 females) studying at The Open University in Hong Kong were recruited by convenience sampling. Age ranged from 18 to 27 years, with a mean of 21.65 years (SD = 2.10). Prospective participants were prescreened for previous history of neurological and mental health problems (e.g., currently taking medication known to affect cognitive performance, cognitive deficits, and diagnosis of PTSD).

### Stimuli and Materials

Suicidal behavior was measured using the Suicidal Behavior Questionnaire-Revised [SBQ-R (66)]. This is a 4-item inventory that explores different dimensions of suicidal thoughts and actions. Item 1 measures lifetime suicide ideation and/or suicide attempts, item 2 assesses the frequency of suicidal thoughts in the previous 12 months, item 3 quantifies the threat of a suicide attempt, and item 4 is the self-reported likelihood of future suicidal behavior. Each question was answered using a Likert scale and the scale for each question differed slightly, with scales ranging from a minimum of 1 to a maximum of 6. Total scores, ranging from 3 to 18, represent overall suicide risk whereby higher scores represent greater risk. In an undergraduate student population the SBQ-R has demonstrated good internal reliability with Cronbach’s alpha ranging from 0.76 (66) to 0.8 (67, 68). Individuals scoring a total of 7 or above were considered to be at a significant risk of suicidal behavior. A cut-off of 7 was selected on the basis of past findings from Osman et al. (66) who found a total score of 7 was most effective at distinguishing between those who had suicide ideation and/or had made a suicide attempt from those who had not experienced suicide behavior. This differs from clinical populations, and while Osman et al. (66) suggest a cut-off of 8, Rueda-Jaimes et al. (69) propose a cut-off of 11 for clinical populations.

The Stroop tasks were presented on a 19˝ computer monitor using E-Prime. In the color Stroop task the words “red,” “yellow,” “blue,” and “green” were presented in bold Times New Roman font, size 28. Each word was presented in the color red, yellow, blue, or green depending on the congruence of the trial. The emotional Stroop task was adapted from Herrington et al. (62) and consisted of positive, negative, and neutral words presented in one of the four colors (red, yellow, blue, and green). A total of 192 words were used from the Affective Norms for English Words [ANEW (70)], 64 positive (e.g., birthday, laughter, angel), 64 negative (e.g., bankrupt, suicide, funeral), and 64 neutral (e.g., handle, carpet, time). Valence of positive words ranged from 6.17 to 8.43 with a mean of 7.49, valence of negative words ranged from 1.61 to 3.69 with a mean of 2.47, and valence of neutral words ranged from 4.02 to 7.57 with a mean of 5.64.

Depression was measured using the Beck Depression Inventory-II [BDI-II (71)]. This self-report inventory measures different aspects of depression such as sadness and irritability. It is a 21-item inventory and all items are assessed on a 4-point rating scale from zero to three (0 indicates no symptoms and a score of 3 indicates severe symptoms). Each item focuses on a particular feeling or behavior and respondents are asked to indicate the extent to which they have experienced this in the previous two weeks. For instance, item 14 focuses on “worthlessness” with responses from 0 (“I do not feel I am worthless”) to 3 (“I feel utterly worthless”). The total score ranges from 0 to 63 with higher scores indicating more severe depression symptoms. A score of 17 or above represents a risk of clinical depression, and scores higher than 31 are indicative of more severe depression. In the current investigation responses to item 9 were removed. This item refers to suicidal behavior and was removed to avoid any overlap with the SBQ-R.

Electroencephalogram activity was recorded using an Emotive EEG Neuroheadset with a sampling rate of 128 Hz (Emotiv Technology Inc., USA) that records from 14 sites (AF3, AF4, F3, F4, FC5, FC6, F7, F8, T7, T8, P7, P8, O1, O2) using a 16-channel Biosemi Active Two system. Two additional electrodes situated at the back of the ears (CMS, DRL) were selected as the reference of choice for all analyses and all sites were referenced to the average of these electrodes during recording, and re-referenced offline. Frontal electrodes were F3, F4, F7, F8, AF3, and AF4. Central electrodes were FC5, FC6, T7, and T8. Parietal electrodes were P7 and P8 and occipital electrodes were O1 and O2. The numbers also indicated the area of the right/left hemispheres of the brain an electrode was located, where even numbers represent the RH and odd numbers refer to the LH. Prior to use, all felt pads on top of the sensors were moistened with a saline solution.

### Procedure

After providing written informed consent participants were seated in a dimly lit room and the EEG headset was affixed to the scalp with sites located according to the 10/20 system (72). The impedance at each site was checked to ensure good contact quality (large signal to noise ratio). Participants were instructed to remain seated in a relaxed state and EEG recordings were taken with the eyes closed for 2 min and the eyes open for 2 min to provide a resting state measure. Next, participants were asked to complete the color and the emotional Stroop tasks while wearing the EEG headset. The order of the tasks was counterbalanced across participants. For both tasks, a trial began with a fixation cross of 500 ms followed by the presentation of a word in the center of the computer screen. For each word participants were asked to identify the color of the text (red, yellow, blue, or green) as quickly as possible by pressing the corresponding key on the computer keyboard (R, Y, B, and G). A total of 60 trials were completed in the color Stroop task with 30 congruent and 30 incongruent trials. There were an equal number of words presented in red, yellow, blue, and green and all trials were presented in a random order. The emotional Stroop task consisted of three emotional blocks showing positive, negative, and neutral words. The order of the blocks was randomized and there were 64 trials in each block. An equal number of words were presented in each of the four colors within each block and all trials were presented in a random order.

### EEG Data Processing

Activity was recorded across the entirety of each block to allow for a general pattern of hemispheric asymmetry to be gained. Consequently activity was taken for all elements within a trial (fixation, stimulus presentation, and response) and a precise measure of electrocortical activity in specific time epochs was not generated. Activity within each block was compared with a baseline measure taken over a period of 20 s in eyes-open resting state immediately prior to each block. All artifact screening, re-referencing, and spectral analysis were performed using EEGLAB toolbox (73) and custom scripts in MATLAB (74). Each data file was visually inspected to manually remove artifacts such as aberrant signals due to large non-blink eye movements, muscle movements, or signal discontinuities. Further EEG artifacts were removed using an independent component analysis [ICA; (73)] during offline signal processing. A bandpass filter of 2–45 Hz and a notch filter of 50 Hz were applied to the raw data with 128-Hz sampling frequency per channel. A Hamming window (1024 sample and 50% overlap) was also applied to the data in preparation for spectral analysis, from which the power and asymmetry estimates were derived.

The experiment was completed in blocks (eyes open resting, eyes closed resting, color Stroop, positive, negative, and neutral emotional Stroop) and activity was recorded and analyzed across each block. Frontal alpha asymmetry was calculated in 1-Hz frequency bins and averaged across the frequency bandwidths of interest: delta (1.5–3.5 Hz), theta (4–7.5 Hz), alpha (8–13 Hz), alpha1 (8–10 Hz), alpha2 (10–13 Hz), beta1 (13–20 Hz), and beta2 (20–28 Hz). Frontal alpha asymmetry was calculated for F3 (left frontal) and F4 (right frontal) electrodes using the Fast Fourier Transform (FFT) method. The alpha power values for F3 and F4 were natural log transformed (73) such that an asymmetry score comparing activity in the RH to activity in the LH in each block was computed [ln ALPHA = (ln[RH] − ln[LH])]. Frontal asymmetry indices were calculated by subtracting the natural log of the power of the LH electrode from that of the RH electrode [ln [right (F4)] − [left (F3)]] (75). Given the inverse relationship between alpha power and cortical activity (76), a positive alpha asymmetry index reflects relatively higher left frontal activity and lower right frontal activity, and a negative asymmetry index reflects relatively higher right frontal activity and lower left frontal cortical activity.

## Results

Two participants were excluded from the analysis due to poor EEG data or missing behavioral data. The remaining 52 participants (31 females) were all right handed and were not taking any medication known to affect brain activity or cognitive performance. The SBQ-R had a suitable level of internal reliability that was consistent with past studies [e.g., Ref. (66)], Cronbach’s α = 0.74. Participants were separated into high and low suicidal behavior groups according to their total score on the SBQ-R. Participants with a total score less than 7 were categorized as low-risk (median score = 5, range = 3–6), while participants with a score of 7 or above were categorized as high-risk (median score = 9.5, range = 7–15). There were a total of 22 participants (13 female, aged 20–23, mean age of 21.55) in the high-risk group and 30 (18 female, aged 20–25, mean age of 21.94) in the low-risk group. Six participants in the high-risk group and none of the participants in the low-risk group reported a past suicide attempt. To ensure that any group differences were not driven by those who had made a past suicide attempt, the results were analyzed once with all participants included, and a second time without attempters. Analysis without attempters is only reported where the results differed from that of the full sample.

The data did not meet parametric assumptions, and therefore a Mann–Whitney *U* test was used to confirm that the SBQ-R scores between the two groups were significantly different (*U* = 0.001, *z* = −6.162, *p* < 0.001, *r* = 0.85). Analysis of scores from the BDI-II (without item 9) also showed that participants in the high suicidal behavior group reported significantly higher levels of depression (median score = 28, range = 17–42) than those in the low suicidal behavior group [(median score = 8, range = 1–16), *U* = 112.500, *z* = −4.034, *p* < 0.001, *r* = 0.57].

### Resting EEG

To investigate differences in alpha-asymmetrical activation in relation to suicidal behavior in the resting state, independent *t*-tests were conducted with suicidal behavior group as the between-participant variable and alpha-asymmetrical index as the dependent variable. Opposite to what was expected, the alpha-asymmetrical index was higher in the high-risk group (*M* = 0.07uV^2^, SD = 0.49) than the low-risk group (*M* = 0.51uV^2^, SD = 0.67) during the eyes-open resting state [*t*(50) = −2.63, *p* = 0.01, Cohen’s *d* = 0.75]. This means that while both groups showed more activity in the left compared with the right, this was most pronounced for the high-risk group. There was no significant difference in alpha asymmetry between the high- and the low-risk groups during the eyes-closed resting state [*t*(50) = −4.497, *p* = 0.141, Cohen’s *d* = 0.42].

### Performance in the Stroop Task

All incorrect trials were removed and any correct response times that were more than 2.5 SD from the mean were classed as outliers and removed (a total of 4.84% of trials). Accuracy was analyzed using a generalized estimating equation (GEE) assuming a negative binomial distribution. All RTs were log transformed to ensure data met the assumptions of a normal distribution and RT data were analyzed using a 2 (suicidal behavior) × 2 (congruency) mixed measures ANOVA.

**Figure 1:**
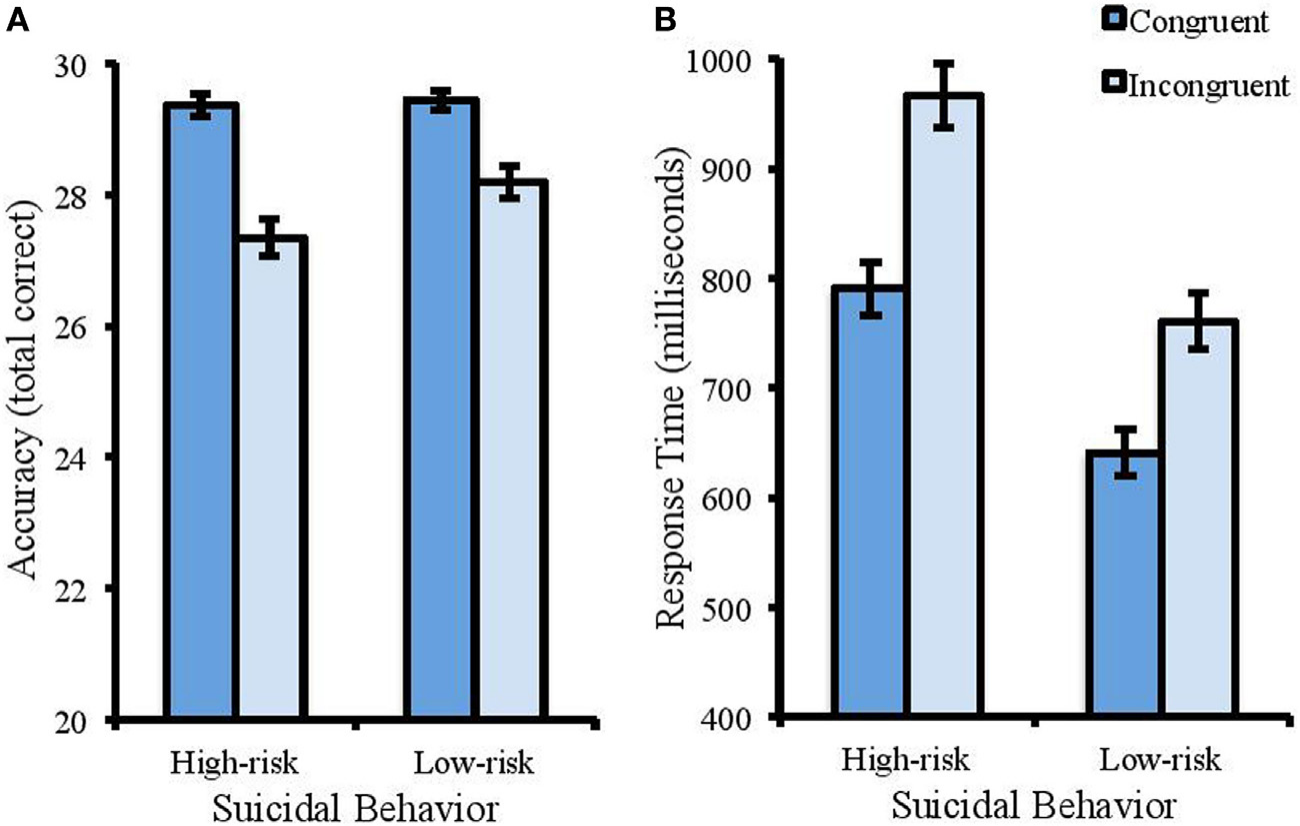
Accuracy (total correct) and RT (ms) in the color Stroop task. Error bars represent standard error of the mean. **(A)** The interaction between suicidal behavior and congruency for accuracy. Reduced accuracy to incongruent trials compared with congruent trials was more apparent for the high-risk group. **(B)** Participants with a high risk of suicide were slower to identify the color of the words (regardless of congruency) than the low-risk group. Response times were also slower to incongruent trials compared with congruent.

Analysis of accuracy in the color Stroop task showed a significant main effect of suicidal behavior [Wald χ^2^ (1) = 4.385, *p* < 0.05, Cohen’s *d* = 0.61]. Accuracy was higher for the low-risk group (*M* = 28.82, SD = 1.16) compared with the high-risk group (*M* = 27.35, SD = 1.71). There was also a significant effect of congruency [Wald χ^2^ (1) = 24.053, *p* < 0.001, Cohen’s *d* = 1.86], with higher accuracy in congruent (*M* = 29.4, SD = 0.76) compared with incongruent trials (*M* = 27.78, SD = 1.51). There was a significant interaction between suicidal behavior and congruency [Wald χ^2^ (1) = 6.158, *p* < 0.05, Cohen’s *d* = 0.73]. Differences between the low- and high-risk groups were only found in the incongruent trials (M of 28.19 and 27.35 respectively, SD of 1.12 and 1.76) and not the congruent trials (M of 29.44 and 29.36 respectively, SD of 0.89 and 0.67) (see Figure 1A).

For RT (see Figure 1B), there was a significant effect of suicidal behavior [*F*(1, 50) = 28.916, MSE = 27712.152, *p* < 0.001, partial η^2^ = 0.366]. Participants reporting lower levels of suicidal behavior showed faster response times than those with higher levels (M of 700.95 and 878.72 ms respectively, and SD of 146.60 and 144.17). There was a significant effect of congruency [*F*(1, 50) = 157.325, MSE = 4360.631, *p* < 0.001, partial η^2^ = 0.719], with faster response times to congruent (*M* = 704.17 ms, SD = 135.59) than incongruent trials (*M* = 848.05 ms, SD = 170.78). There was no interaction between suicidal behavior and congruence [*F*(1, 50) = 1.222, MSE = 4360.63, *p* = 0.274, partial η^2^ = 0.084].

There was no significant difference in alpha-asymmetrical index between the high-risk group (*M* = 0.334uV2, SD = 0.50) and the low-risk group (*M* = 0.452uV2, SD = 0.85) in the color Stroop task [*t*(50) = −0.580, *p* = 0.564, Cohen’s *d* = 0.18].

### Performance in the Emotional Stroop Task

Analysis of the emotional Stroop task followed that of the color Stroop task. Accuracy was analyzed using a GEE and RT was analyzed with a 2 (suicidal behavior) × 3 (emotion) mixed measures ANOVA. A total of 4.21% of trials were removed due to low accuracy or because response times were more than 2.5 SD from the mean. All RTs were log transformed to satisfy distributional assumptions.

**Figure 2:**
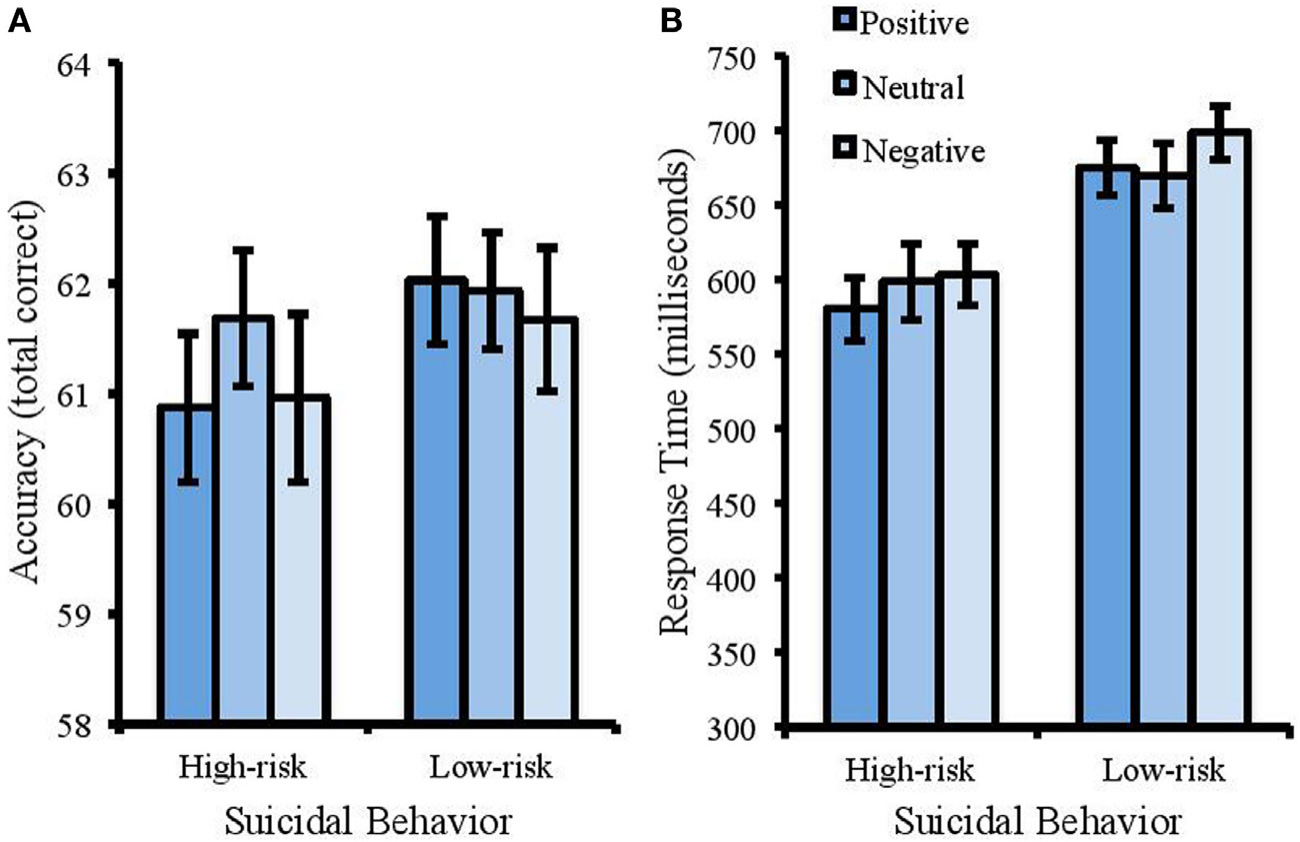
Accuracy (total correct) and response times (ms) in the emotional Stroop task. Error bars represent standard error of the mean. There was a speed-accuracy trade-off in this task whereby the high-risk group responded faster **(B)** but were less accurate **(A)**.

For accuracy (Figure 2A), the model revealed a significant main effect of suicidal behavior [Wald χ^2^ (1) = 4.069, *p* < 0.05, Cohen’s *d* = 0.58]. Accuracy was higher for the low-risk group (*M* = 61.88, SD = 2.39) compared with the high-risk group (*M* = 61.17, SD = 1.74). There was no main effect of emotion [Wald χ^2^ (1) = 1.034, *p* = 0.309, Cohen’s *d* = 0.28], and no interaction between suicidal behavior and emotion [Wald χ^2^ (1) = 2.483, *p* = 0.115, Cohen’s *d* = 0.45].

For RT (Figure 2B), there was a significant effect of suicidal behavior [*F*(1, 50) = 11.30, MSE = 8495.123, *p* < 0.001, partial η^2^ = 0.184] with faster response times in the high-risk group (*M* = 593.31 ms, SD = 84.83) compared with the low-risk group (*M* = 679.76 ms, SD = 94.95). There was no significant effect of emotion [*F*(2, 100) = 1.824, MSE = 3969.585, *p* = 0.110, partial η^2^ = 0.035] and no interaction between suicidal behavior and emotion [*F*(2, 100) = 0.608, MSE = 2413.522, *p* = 0.546, partial η^2^ = 0.012].

To assess inhibition of suicide-related stimuli, response times were also considered across the two groups when responding to the word “suicide.” A between-participants *t*-test was conducted that showed significantly longer response times for the high-risk group compared with the low-risk group [M of 725.20 and 652.78 respectively, SD of 126.32 and 116.12, *t*(50) = 2.17, *p* = 0.035, Cohen’s *d* = 0.6].

**Figure 3:**
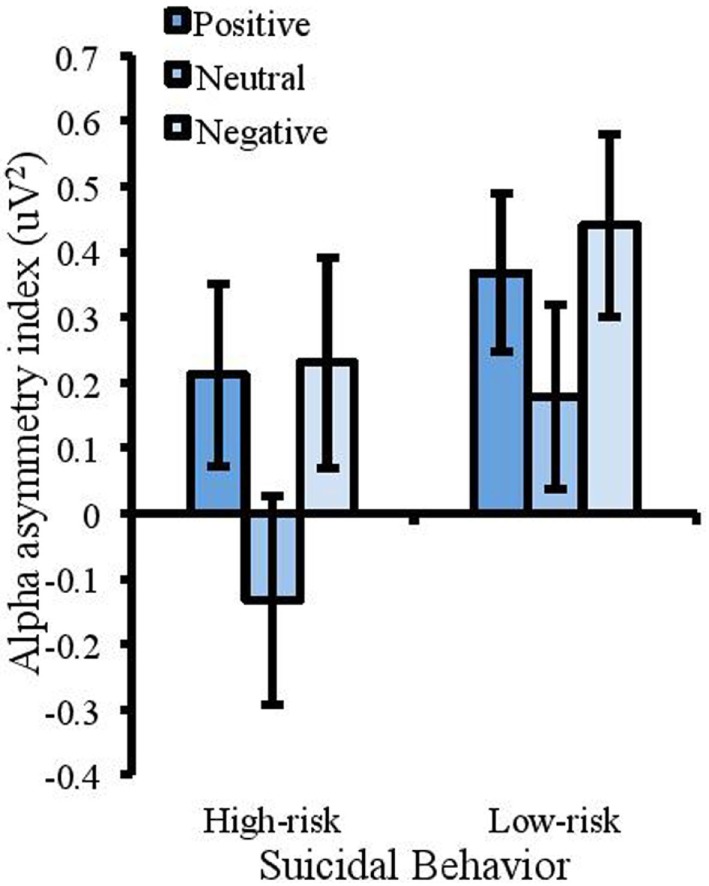
Measures of frontal alpha asymmetry (uV^2^) in the emotional Stroop task showing a more positive index for the low-risk group (a positive alpha asymmetry index reflects lower right frontal cortical activity and a negative asymmetry index reflects lower left frontal cortical activity). The asymmetry index was also higher for emotional trials compared with neutral. Error bars represent standard error of the mean.

A 2 (suicidal behavior) × 3 (emotion) mixed measures ANOVA was conducted to analyze alpha-asymmetrical index in the emotional Stroop task (where sphericity was violated Greenhouse–Geisser corrections are reported). This showed a significant effect of suicidal behavior [*F*(1, 50) = 4.024, MSE = 0.484, *p* = 0.05, partial η^2^ = 0.074], with a more positive index in the low-risk group (*M* = 0.49uV^2^, SD = 0.81) compared with the high-risk group (*M* = 0.10uV^2^, SD = 0.61). With the removal of attempters from the high-risk group, this effect was no longer significant [*F*(1, 44) = 2.955, MSE = 0.501, *p* = 0.093, partial η^2^ = 0.063]. There was also a significant effect of emotion [*F*(1.358, 67.91) = 13.73, MSE = 0.113, *p* < 0.001, partial η^2^ = 0.215]. Planned contrasts were completed to compare asymmetry in the positive and negative conditions to that in the neutral condition. These revealed that the alpha-asymmetry index was significantly higher for negative words (*M* = 0.44uV^2^, SD = 0.78) compared with neutral words [(*M* = 0.18uV^2^, SD = 0.80), *F*(1, 50) = 16.632, MSE = 0.231, *p* < 0.001, partial η^2^ = 0.250] and higher for positive words (*M* = 0.37uV^2^, SD = 0.66) compared with neutral words [*F*(1, 50) = 12.852, MSE = 0.175, *p* = 0.001, partial η^2^ = 0.204]; see Figure 3. There was no interaction between emotion and suicidal behavior [*F*(1.358, 67.91) = 3.068, MSE = 0.113, *p* = 0.072, partial η^2^ = 0.058]. This interaction was however significant when attempters were removed from the high-risk group [*F*(1.38, 60.902) = 5.312, MSE = 0.079, *p* < 0.05, partial η^2^ = 0.11]. This supported a trend showing that the high-risk participants showed a negative asymmetry index in the neutral condition compared with the positive [*F*(1, 44) = 7.724, MSE = 0.185, *p* < 0.01, partial η^2^ = 0.15] and negative conditions [*F*(1, 44) = 4.565, MSE = 0.232, *p* < 0.05, partial η^2^ = 0.094]. This reflects more rightward relative to leftward activity in the neutral condition and this pattern was not found for the low-risk participants.

## Discussion

Deficits in cognitive processing and neurological activity have been consistently linked to suicidal behavior in previous research (7, 9, 24) and the current study sought to extend this work by examining the association between frontal asymmetry, attentional control, and suicidal behavior. Frontal asymmetry was compared between individuals reporting high and low levels of suicidal behavior at resting state (both eyes closed and eyes opened), during a color Stroop task, and during an emotional Stroop task. It was predicted that individuals with a high risk of suicidal thoughts and actions would show general difficulties in attentional control, difficulties inhibiting negative stimuli, and reduced leftward-frontal activity.

In the color Stroop task, the high-risk group took significantly longer and were less accurate than the low-risk group to identify the color of each word, regardless of whether this was congruent or incongruent. They were also less accurate when responding to incongruent trials. This shows their difficulties with inhibiting irrelevant information. The results are consistent with previous research [e.g., Ref. (16, 26)], showing that suicidal individuals have more difficulty inhibiting distracting information. Inhibition is one of three components of executive function (15) that contributes to the control and regulation of behavior. It is a crucial element within attentional control and in many every-day tasks an individual needs to inhibit the automatic processing of irrelevant information and direct attention toward relevant information. It is argued that poor attentional control contributes to suicidal behavior as it prevents the disengagement from suicide-related thoughts making one less able to resist suicidal urges (26), and it limits the redirection of resources to more positive information therefore maintaining negative biases.

In contrast to the results of the color Stroop task, in the emotional Stroop task the high-risk group responded quicker than the low-risk group (although this was at the expense of accuracy). This pattern was found for all three types of words (positive, negative, and neutral) and would indicate that those reporting high levels of suicidal behavior are able to inhibit irrelevant information more effectively than those reporting low levels. The overall lack of any emotional Stroop effect within this task is also inconsistent with past findings showing that response times in an emotional Stroop task are generally slower to emotional words compared with neutral words (34, 35). It may be proposed that individuals with a high risk of suicidal behavior are slower to inhibit irrelevant information at a general level, yet when presented with emotional stimuli they may act more quickly and somewhat impulsively (this would be supported by the speed-accuracy trade-off whereby the high-risk group sacrificed accuracy for faster responses). The importance of impulsivity has been identified in the warning signs for suicidal behavior listed by the American Association of Suicidology and includes acting recklessly (77). The Association documented that the presence of impulsivity, inhibitory problems, and inflexible thinking processes may lead to an increased risk of suicidal behavior. Rudd (78) has also incorporated the measures of impulsivity into suicide risk assessment tools. This reflects the proposed importance of executive dysfunction in suicidal behavior with symptoms indicative of poor updating (sustained focus on negative information), shifting (inability to direct resources to task-relevant information), and inhibition (inability to suppress the processing of irrelevant, negative information).

While it may be argued that those with high levels of suicidal behavior can respond more quickly to emotional stimuli compared with neutral, one may question why this group did not show longer response times in the neutral condition of the emotional Stroop task (similar to the color Stroop). This effect illustrates key differences between these two tasks. In particular, in a color Stroop the to-be-ignored information in incongruent trials (the word) is in direct competition with the to-be-identified information (the color). This is not the case in the emotional Stroop task. Consequently the differing patterns of performance across the two tasks may indicate that individuals with a greater risk of suicide will have more difficulty inhibiting directly competing responses, but not information that has no semantic relationship to the task they are completing. Further support for this argument comes from the response times in identifying the color of the word “suicide” in the emotional Stroop task. Results showed that the pattern of performance in the task reversed and those in the high-risk group took longer to respond to the color of this word showing that they have difficulties inhibiting emotionally relevant information. It is proposed that such “personally” relevant information is more salient and despite being irrelevant to the task it competes for attentional resources in the same way that the directly competing word meaning does in the color Stroop.

The bias of attention to emotionally significant stimuli supports the findings of Chung and Jeglic (79) who also reported no emotional Stroop effect in individuals high in suicidal behavior but found evidence for a specific attentional bias to the word “suicide.” Cha et al. (80) propose that a stimulus-specific Stroop interference effect (whereby only disorder-related words lead to longer response times) may be particularly useful for clinicians. They found that it was able to predict, above and beyond other clinical measures, those individuals who went on to make a suicide attempt within the following 6 months. Evidently, the current findings support this suggestion, in which a specific attentional bias may exceed the predictive ability of any general negativity bias. This can add to cognitive models that attempt to explain the development and persistence of affective disorders such as depression [e.g., Ref. (17, 18)]. It is theorized that an individual will be automatically distracted by negative information and the processing of this information will lead to an attentional bias. The results of the emotional Stroop task would suggest that these biases are disorder-specific, and while general deficits in top–down control predicted in the model will limit inhibitory processing at a general level (as demonstrated in the Stroop task) it will also manifest in specific impairments in the ability to inhibit disorder-related thoughts and behaviors.

In addition to measuring the importance of attentional control in suicidal behavior, the current study also aimed to determine whether patterns of frontal asymmetry could be used to identify those at risk of suicidal behavior. The dispositional model (45, 46) argues that positive affect is associated with leftward frontal cortical activity and negative affect is associated with rightward frontal cortical activity, whereas the capability model (57) predicts that frontal asymmetrical differences will be more pronounced under specific situational contexts [(57); see also Ref. (81)]. To examine frontal asymmetry in relation to both models, activity was measured during an emotionally challenging state (the emotional Stroop) to see if this may provide a more promising indicator of suicide risk than activity measured during resting state (as favored by the dispositional model) and during a challenging but non-emotional task (the color Stroop).

The EEG recordings in the eyes closed resting state gave no support for the dispositional model as individuals with high and low risk did not differ in their alpha asymmetry index. Although there was a significant group difference during the eyes open resting condition, the difference was opposite to the predictions made. Individuals in the high-risk group had more leftward frontal activity than the low-risk group indicating that this side of the brain is more active at baseline. It is interesting to note that increased leftward frontal activity is associated with the inhibition of negative information (60) and may reflect inhibition of general negative thoughts that an individual with suicidal behavior could be experiencing when not completing a demanding task (this would not be apparent in the low-risk group as it is predicted they would not experience upsetting thoughts and so would not need to engage in inhibition). However, when the EEG recordings were taken during the color Stroop task there was no significant difference in alpha-asymmetrical index between high- and low-risk groups. This reveals that measurements of frontal asymmetry taken during a demanding task are no more effective than those taken in a resting state with regard to identifying individuals high in suicidal behavior. The differences between activity in the Stroop and the eyes open resting state may also suggest that when engaged in a demanding neutral task high-risk participants in the current sample (reporting relatively mild suicidal behavior) are not having to devote additional resources to the inhibition of negative thoughts because the focus on the task itself prevents the processing of such information. Yet the results of asymmetry do not reflect performance in the Stroop task as the high-risk group performed less well than the low-risk group but showed no corresponding differences in frontal asymmetry. This may be due to the fact that stimuli in this task were neutral and therefore any increase in activity is unlikely to be related to specific inhibition of positive (right) or negative (left) information. This indicates a limitation to the use of asymmetry as a marker for affective disorders, including suicide. Compton et al. (28) found increased activity overall in the dlPFC for incongruent trials in a color Stroop and suggested that this shows greater investment in cognitive control processes in order to inhibit this information. Asymmetry does not provide a direct measure of activity and instead shows relative differences between the left and right. Arguably the measure is more relevant to the processing of emotional information if left and right areas are associated with inhibition of negative and positive stimuli respectively.

Consistent with proposals of the capability model, the results did reveal a significant difference in frontal asymmetry between the high- and low-risk groups during the emotional Stroop task. In particular, the low-risk group showed more leftward frontal activation compared with the high-risk group. This suggests greater recruitment of left frontal areas during completion of a task that requires inhibition of emotional information (although not specifically negative information as the models of asymmetry suggest). It should be noted that this effect disappeared when individuals reporting a past suicide attempt were removed from the analysis suggesting that the effect was driven by this subset of participants. This is supported by the findings of Jollant et al. (59) in which asymmetrical differences were only found in individuals with a history of suicide attempt. While behavioral performance in the Stroop tasks may be able to distinguish those at risk of mild levels of suicidal behavior (and would therefore be beneficial in identifying those at risk at an early stage) the same may not be concluded for measures of alpha asymmetry. In addition, the findings for asymmetry do not reflect performance in the task because those in the high-risk group were faster to make accurate responses. Once again this may indicate the limitations of using asymmetry as a marker because it reflects relative activity, it does not show whether an individual is putting more effort overall into the task. A study by Kaiser et al. (82) showed increased activation in the dorsal anterior cingulate cortex and the posterior cingulate cortex for depressed patients when completing a task requiring the inhibition of negative distracters. They proposed that increased activity demonstrates that individuals are devoting more cognitive resources to directing attention away from negative information; however, frontal asymmetry does not provide information about such overall patterns of activity.

Grimshaw and Carmel (60) suggest that inhibition of different emotional stimuli is linked to frontal alpha asymmetry and that individuals will exhibit leftward frontal cortical activity during inhibition of negative stimuli, and rightward frontal cortical activity during inhibition of positive stimuli. Although there is considerable evidence to suggest that frontal asymmetry reflects the inhibitory control of emotions [e.g., Ref. (60, 83); see Ref. (84)], the current findings provide only partial support for the asymmetric inhibition model. Individuals were showing more leftward frontal activation during inhibition of negative stimuli as predicted; however, they did not show an increase in rightward frontal activity when inhibiting positive stimuli. These results are similar to past findings (60, 62) that have shown that the links between cortical activity in the right dlPFC and control of positive distractors are different to those between the left dlPFC and the control of negative distractors. For example, Pérez-Edgar et al. (83) conducted a study investigating frontal asymmetry in relation to attentional bias and avoidance. Frontal EEG was measured from young adults at rest and under a socially threatening situation (preparing to give a short speech about their most embarrassing moment in public). Following this, participants performed a dot probe task in which they had to respond to probes appearing in the same spatial location as emotional faces. Results showed that although frontal alpha asymmetry in the resting state did not predict performance in the dot probe task, there was a strong link between behavioral performance and frontal asymmetry in the socially threatening condition. Specifically, an increase in rightward frontal alpha asymmetry in this condition was associated with increased attentional bias to angry faces and avoidance of happy faces but no association between leftward frontal asymmetry and emotions. This trend was replicated by Grimshaw and Carmel (60) who suggested that positive and negative stimuli may not exert the same level of influence on frontal alpha asymmetry.

One unexpected finding from the alpha asymmetry analysis was the trend toward a negative alpha asymmetry index in the neutral condition of the emotional Stroop task for the high-risk group. This trend did not reach significance until participants reporting a past suicide attempt were removed from the analysis, but the pattern of activation was markedly different to that of the other conditions. The finding shows that the high suicidal behavior group had relatively lower leftward activation in the neutral condition suggesting that they only recruited more left frontal areas when inhibiting emotional but not neutral information. Again, this was not evidenced by differences in performance in this task, providing limiting support for the use of asymmetry as a marker of suicidal behavior, and showing that the exact role of the right and left PFC is not yet apparent with regard to the inhibition of positive and negative distracters. Furthermore, Gable et al. (84) proposed that frontal asymmetry may reflect a wide range of cognitive mechanisms, not just inhibitory processes. For example, the dlPFC is activated during tasks requiring task switching (85), working memory (86), emotion regulation [for a review, see Ref. (30)], and attentional disengagement (87). All of these are implicated in vulnerability to psychopathologies associated with frontal asymmetry (88). These processes also require the executive control components of updating and shifting in addition to inhibition (10, 15, 89). Future work would benefit from recording performance and activity in a wider range of neuropsychological tasks [i.e., Ref. (90)].

The present results show some support for the association between attentional control, frontal asymmetry, and suicidal behavior. However, the findings do not fully support previous work and therefore may indicate that other factors may be involved. In particular, the current results may be influenced by depression. A measure of depression was taken from all participants and analysis showed clear differences between the two groups with the high-risk group reporting significantly higher symptoms of depression. It is well documented that depression is comorbid with suicide [e.g., Ref. (90)] and studies provide strong evidence for the links between depression and executive dysfunction [e.g., Ref. (10)] and depression and frontal asymmetry [e.g., Ref. (41)]. Consequently, the present findings may be showing differences due to depression, rather than suicide. However, researchers argue that the Stroop task is one of very few measures of executive control that is able to identify differences between levels of depression and suicidal behavior. Richard-Devantoy et al. (90) conducted a meta-analysis to explore the findings of studies investigating executive control in patients with mood disorders, patients with mood disorders and reporting a past suicide attempt, and healthy controls. Across a number of tasks designed to assess executive function they found that the patients performed worse than the healthy controls, yet performance in the Stroop task was also able to distinguish suicide attempters from non-attempters. Given the differences between the two groups in the color Stroop task, and the fact that the high-risk group showed a specific attentional bias to suicide-related information, rather than a general negativity bias [e.g., Ref. (37)], it is argued that the present study is assessing suicidal behavior additional to the effects of depression.

While it may be argued that this study assesses suicidal behavior, the results are limited due to the use of the SBQ-R (66). This is a relatively simplistic single-item assessment that groups a variety of quite distinct suicidal behaviors together. Many past studies in this field utilize more in-depth assessments and often use a mixture of clinical measures and interviews. Milner et al. (91) express concern over the use of single-item assessments due to the increased risk of Type-I and -II errors and after conducting an evaluation of such measures they found that many were unable to capture the precise nature of suicide-related thoughts and behaviors that were reported. While these limitations are acknowledged and future research would make use of more detailed measures, it is important to note that the aim of this study was to measure the association of attentional control, asymmetry, and suicidal behavior, rather than to measure whether deficits varied according to the severity of symptoms. The SBQ-R has benefits in this case due to the relative ease of administration.

Related to the measurement of suicide, future studies that explore variations in attentional control due to severity of suicidal behavior may employ a correlational design to allow for the prediction of suicide through measures of executive control. The small sample size and the relatively limited spread of suicidal behavior in the current study supported the use of group comparisons but arguably the findings have no predictive power. Given that past research focuses on more clinical samples, and often uses older patients [e.g., Ref. (26)] one key feature of the present work was to explore possible cognitive deficits associated with relatively mild symptoms of suicidal behavior. By showing that suicidal behavior in a non-clinical population is associated with deficits in attentional control (specifically difficulties inhibiting irrelevant information and an attentional bias to emotionally pertinent information) the current work expands on the past studies. For instance, when comparing executive function in depressed suicide attempters, depressed non-attempters, and healthy controls Keilp et al. (16) supported the findings of Richard-Devantoy et al. (90) by showing that performance in a Stroop task was a “relatively independent marker of suicide risk” (p546). In their study, deficits in attentional control (as evidenced through the Stroop task) were found in all individuals with a history of suicide attempt. In the current study the comparison of attempters and non-attempters was not possible as only 6 of those in the high-risk group reported a past suicide attempt, yet performance in the Stroop task did identify those more vulnerable to suicidal thoughts and behaviors. The findings demonstrate the effectiveness of the Stroop task in assessing vulnerability to suicide in non-clinical samples and support its use in the intervention and prevention of suicidal behavior.

Using EEG in a color Stroop task and an emotional Stroop task, the current study examined whether measures of cognitive and neurological processing can be used to identify individuals at risk of suicidal behavior. The study compared attentional control and frontal asymmetry between individuals reporting high and low levels of suicidal behavior. Results showed that individuals reporting higher levels of suicidal behavior are more likely to encounter difficulties in attentional control and will struggle to disengage attention from suicide-related information. The findings provide relatively limited support for the effectiveness of frontal asymmetry in identifying those vulnerable to suicide, and in line with the capability model of Coan et al. (57) general differences were only apparent in the emotional Stroop task. By exploring executive dysfunction in a non-clinical sample reporting relatively mild symptoms of suicidal behavior the current work lends support to those who advocate the use of the Stroop task in prevention of suicide, showing that its effectiveness extends beyond patient groups.

## Ethics Statement

This study was carried out in accordance with the recommendations of The British Psychological Society. The protocol was approved by the Research Ethics Panel for the School of Health Sciences at the University of Salford. All participants gave written informed consent in accordance with the Declaration of Helsinki.

## Author Contributions

CT and EO conceived and designed the study. EO gained ethical approval and collected the data. CT and EO analyzed the data and drafted the manuscript.

## Conflict of Interest Statement

The authors declare that the research was conducted in the absence of any commercial or financial relationships that could be construed as a potential conflict of interest.
